# Melatonin: Bone Metabolism in Oral Cavity

**DOI:** 10.1155/2012/628406

**Published:** 2012-08-09

**Authors:** Fanny López-Martínez, Patricia N. Olivares Ponce, Miriam Guerra Rodríguez, Ricardo Martínez Pedraza

**Affiliations:** Department of Advance General Dental Master, School of Dentistry, Universidad Autónoma de Nuevo León, Hidalgo 2425-403, Col. Obispado, Monterrey, NL, Mexico

## Abstract

Throughout life, bone tissue undergoes a continuous process of resorption and formation. Melatonin, with its antioxidant properties and its ability to detoxify free radicals, as suggested by Conconi et al. (2000) may interfere in the osteoclast function and thereby inhibit bone resorption, as suggested by Schroeder et al. (1981). Inhibition of bone resorption may be enhanced by a reaction of indoleamine in osteoclastogenesis. That it has been observed melatonin, at pharmacological doses, decrease bone mass resorption by suppressing through down regulation of the RANK-L, as suggested by Penarrocha Diago et al. (2005) and Steflik et al. (1994). These data point an osteogenic effect towards that may be of melatonin of clinical importance, as it could be used as a therapeutic agent in situations in which would be advantageous bone formation, such as in the treatment of fractures or osteoporosis or their use as, a bioactive surface on implant as suggested by Lissoni et al. (1991).

## 1. Introduction

The bone tissue is a variety of connective tissues that are essentially a mineralized extracellular matrix and specialized cells: osteoblasts, osteocytes, and osteoclasts.

The organic component, or osteoid matrix produced by osteoblasts, is constituted by 90% of type I collagen fibers, which represents the major structural protein of bone matrix. The remaining 10% is made up of a series of non-collagenous proteins that modulate smaller mineralization and binding of cells to the matrix, and among them (see Table 1). 

The inorganic phase consists of small crystals of a mineral alkaline character, hydroxyapatite [Ca_10_(PO_4_)_6_(OH)_2_]. These crystals are embedded among the collagen fibers to form a fabric that meets the appropriate characteristics of rigidity, flexibility, and endurance [1–3].

Osteoblasts derived embryologically multipotent progenitor cells from bone marrow stroma. These cells originate from osteoblasts, as well as fibroblasts, chondrocytes, adipocytes, and muscle cells, some of which are phenotypic characteristics similar to those of osteoblast. These cells are metabolically active secretory proteins expressed as osteocalcin and osteopontin, osteonectin and other proteoglycans and soluble markers factors (BMPs, TGF-*β*, IGF I and II, IL-1 and PDGF).The expression of these products by osteoblasts bone occurs during embryogenesis, and during maintenance (remodeling), and repair.The signals that direct osteoid mineralization have not yet been identified.It is likely that the accumulation of proteins in a calcium will be the beginning of the process of mineralization [4].

Although osteoblasts are polarized towards the bone, the release of the osteoid matrix proteins is not limited to the basal pole, but many of them are being involved in such a matrix, becoming osteocytes included in the gaps that form in it.

Osteoblasts can lead to surface osteocytes, also known as lining cells.Both types of bone cells possess receptors for parathyroid hormone (PTH) and express mRNA Live: *β*-actin, transcription factors c-fos and c-jun proteins collagen and collagen as well as mRNA-like growth factor insulin I (IGF-I) [5].

Already formed in the bone, the osteocytes included in the gaps or osteoplasmas in the mineralized matrix, are star-shaped with numerous thin extensions, and are connected by a network of canals or ducts calcoforos, bathed by the so-called bone fluid.Such networks of channels are formed before they mineralize the osteoid matrix.

When there is bone resorption by osteoclasts, osteocytes are out of the lagoons as cladding in repose cells [3].Bone resorption is a complex process involving the dissolution of the mineral or inorganic phase and subsequent degradation of bone matrix proteins.Osteoclasts adhere to the bone surface via integrins that specifically recognize osteoid matrix proteins.The sealing area delimits a microgap between osteoclasts and bone surface. Demineralization is produced by acidification of the microgap by the action of Hydrogen (H+) ATPaselocated in the brush border membrane.To maintain the physiological pH within it, the osteoclast has a heat exchanger (Cl^−^/HCO_3_^−^)on the opposite side of the brush border and, on this edge, a channel Chlorine (Cl^−^) coupledH^+^-ATPase.As a consequence the osteoblast secretes HCl in the microsubosteoclastic, thus lowering the pH to 4.4 and the dissolution of the mineral.This dissolution precedes the organic matrix degradation, carried out by proteases such as cathepsin K, secreted by osteoclasts, and collagenase by osteoblasts [6, 7].

## 2. Bone Remodeling

Throughout life, bone tissue undergoes a continuous process of resorption and formation.By continuing, there are areas of bone which are destroyed to be replaced by newly formed bone tissue.This process is called “bone remodeling” and leads to the replacement of about 7–10% of the total volume of the skeleton each year.

The remodeling is done by groups of cells, osteoblasts and osteoclasts that are called “units of remodeling” or “basic multicellular units.” The process begins when osteoclast precursors are attracted to a particular location of the bone, and the action of cytokines (TNF-*α*, IL-1, IL-6, M-CSF, RANK-L) differentiates osteoclasts  *in situ*.These begin to reabsorb bone, along untill two weeks later a small cavity is lined by mononuclear cells called the investment phase. After osteoblast precursors are recruited to proliferate and differentiate osteoblasts are arranged in a monolayer to synthesize osteoid filling while the gap is opened by the osteoclasts.It is the bone formation phase, which lasts for 2–4 months.The mineralization of sheets of osteoid is being produced as deposited, but with a delay of 2 weeks.At the end of the process, the result is that a small amount of old bone by new bone renovated [8] (see Table 2). 

Numerous studies documented that melatonin is an important mediator in bone formation and stimulation [9]. At micromolar concentrations, melatonin synthesis stimulates the proliferation and collagen fibers of type I in human osteoblasts *in vitro* [10].

The effect of pineal gland/melatonin (aMT) upon circadian secretion of hormones, especially those playing a crucial role in the regulation of bone metabolism appears significant [11, 12]. Early experimental studies conditions have shown that long-term light,and pinealectomy also modified the synthesis aMT administration and/or circadian release of growth hormone (GH), insulin-like growth factor-I (IGF-I) as well as calciotropic, thyroid, adrenal cortex hormones and testes. Also enhances synthesis of aMT and noncollagenic proteins of bone matrix [10].That characteristic suggests that clinical studies of bone mass changes in postmenopausal osteoporosis may be related to aMT [13].Studies demonstrate that increased obese women aMT secretion in more than 20% overweight have protective significance in loss of bone mass aftermenopause [14].

Melatonin is not a hormone in the classic sense, but as a cell protective functions and an antioxidant [15].It is known that the enzymes required for the biosynthesis of melatonin are found in tissues other than the pineal gland, and it is also known that several of these tissues, amongst them the retina, thymus, spleen, B-lymphocytes, ovaries, testicles, and the intestine, all produce melatonin.Extrapineal melatonin is produced by specific organs used locally as paracoid or autocoid and does not enter the circulation [16].Melatonin does not act upon specific target organ and stock. It reaches all tissues and due to amphiphilicity, it enters to all subcellular compartments [17, 18].Moreover, several organelles and the nucleus including the mitochondria may accumulate melatonin [18, 19].

Melatonin, with its antioxidant properties and its ability to detoxify free radicals [20] in this may interference of the osteoclast function and thereby inhibit bone resorption [21].Inhibition of bone resorption may be enhanced by a reaction of indoleamine in osteoclastogenesis. It has been observed that melatonin, at pharmacological doses, increases bone mass by supressing resorption trough down-regulation of the RANKL-mediated osteoclast formation and activation. These data point an osteogenic effect towards that may be of melatonin of clinical importance, as it could be used as a therapeutic agent in situations in which there would be advantageous bone formation, such as in the treatment of fractures or osteoporosis [21].

Links between melatonin metabolism and bone have been documented in many studies [10, 21, 22]. Investigations, acted melatonin on the bone as a local growth factor, with paracrine effects on nearby cells [23, 24]. 

## 3. Osseointegration in Oral Implantology

Osseointegration requires the formation of new bone around the implant, resulting in the remodeling process within the bone tissue.The process was initiated by osteoclasts, which are the cells responsible for reabsorbing the necrotic area originated by bone drilling during the preparation of the recipient bone.With them, vascular neoformation provide the cells elements, the osteoblasts, which create new bone able to interact with the titanium oxide layer of the implant to integrate biologically to it.Osteoblasts are differentiated osteocytes subsequently included in the mineralized bone matrix.  Considering ultrastructurally bone-implant interface must refer to the concept of biointegration, which is the direct biochemical joint between the bone and the implant surface, demonstrable through electronic microscopy, regardless any interunion mechanism.Thus, the space between the mineralized bone tissue and titanium plasma coating of the implant is not more than 10 Angstrom and appears filled with a matrix material, the proteoglycans [25].A network of collagen is surrounding the osteocytes and inserted into the layer of glycoproteins, which fusions with titanium oxide layer.It is believed that the implant titanium oxide induces the formation of sulfated glycosaminoglycans [26].The bony trabeculae grow closer to the implant contacting the plasma layer.From them come the vessels supplying nutrition, cellular elements for remodeling and around the implant, fibroblasts, and osteoblasts to increase and approach the implant which is attached to the oxide layer.They are fundamental substances that fills the trabecular spaces and fuse with the titanium oxide layer.Therefore, and contrary to what is believed, the oral tissue-implant interface is a dynamic area under intense remodeling activity by the bone cells and the extracellular matrix [27].

## 4. Melatonin and Cancer

Melatonin can kill directly many different types of human tumor cells [28, 29].It is a naturally produced cytotoxin, which can induce tumor cell death (apoptosis) [30, 31]. In instances where the tumor has already established itself in the body, melatonin has been shown to inhibit the tumor's growth rate [32, 33].Melatonin exhibits natural oncostatic activity and inhibits cancer cell growth [34].In patients in whom cancer already has become a noticeable physical burden which produces overt symptoms, melatonin has been shown to alleviate numerous cancer symptoms [35] and to inhibit development of new tumor blood vessels (tumor angiogenesis) [36], which in turn inhibits the cancer from spreading further (metastasis) [37]. Melatonin can retard tumor metabolism and development by lowering the body temperature; it is a natural inducer of hypothermia. Furthermore, as an inducer of antioxidants [38], and itself a weak preventive antioxidant, melatonin hinders tumor cells from participating in free radical damage to normal cells and consequently limits oxidative damage to DNA, lipids, amino acids, and proteins. Melatonin promotes bone formation [39] and on the other hand with its antioxidant action on free radicals interferes with the osteoclast and thus inhibits bone resorption [40, 41].

## 5. Conclusion

Although today the designs and surface treatment of implants are allowing in some cases reduce load times, generally provides a period of osseointegration in the mandible 3 months and 6 months in the maxilla.Cutando et al. [42] found that at 2 weeks of melatonin implants significantly increased all parameters of osseointegration of them: percentage of bone contact, total peri-implant bone, bone interscrew, and the percentage of new bone formation. Roth et al looked melatonin stimulated, the expression of bone, sialoprotein genes, alkaline phosphatase and osteocalcin for a period of time from 5 to 9 days, founding a highly significant evidence for this relationship because the genes of many of the bone matrix proteins (BSP, ALP, OC, and SPARC) contain the sequence of bases (RGGTCA) required for nuclear receptor binding of melatoninRZR to its promoter region. However, this increase in bone tissue formation may also be mediated by membrane receptors of the indoleamine, and that treatment with pertussis toxin and luzindole is able to reduce the expression of BSP and ALP [43].

All these data confirm that an osteogenic effect of melatonin may be clinically important as it could be used as a potential therapeutic agent in situations where it is desirable to increase bone formation and healing of fractures or osteoporosis, or their use as a bioactive surface on implant [44]. 

## Figures and Tables

**Table 1:** Osteoid matrix proteins.

(1) Collagen type I (90%)
(2) Noncollagenous proteins (10%)
(i) Glycoproteins:
(a) Alkaline phosphatase
(b) RGD sequence-glycoproteins (osteopontin, osteonectin, fibronectin, thrombospondin, and bony sialoprotein)
(ii) Proteoglycans
(iii) Proteins with *γ*-carboxyglutamic acid (osteocalcin, osteoid protein *γ*-carboxyglutamic acid)
(iv) Serum proteins retained in the bone

**Table 2:** Regulatory factors of bone modeling.

(i) Hormones
(a) Parathyroid hormone (PTH)
(b) 1, 25 (OH)_2_ vitamin D
(c) Calcitonin
(d) Glucocorticoids
(e) Growth hormone
(f) Sex hormones (estrogens and androgens)
(ii) Local factors (paracrine and autocrine)
(a) Growth factors and cytokines
(iii) Other molecules
(a) Prostaglandins, leukotrienes, extracellular ATP, Bradykinin, CGRP
